# Anti-AMPA-Receptor Encephalitis Presenting as a Rapid-Cycling Bipolar Disorder in a Young Woman with Turner Syndrome

**DOI:** 10.1155/2015/273192

**Published:** 2015-10-01

**Authors:** Giuseppe Quaranta, Angelo Giovanni Icro Maremmani, Giulio Perugi

**Affiliations:** ^1^Department of Experimental and Clinic Medicine, Section of Psychiatry, University of Pisa, Via Roma 67, 56100 Pisa, Italy; ^2^The Institute of Behavioural Science “G. De Lisio”, Via di Pratale 3, 56127 Pisa, Italy

## Abstract

*Background.* Autoimmune encephalitis is a disorder characterised by the subacute onset of seizures, short-term memory loss, and psychiatric and behavioural symptoms. Initially, it was recognised as a paraneoplastic disorder, but recently a subgroup of patients without systemic cancer was identified. *Case Description.* We describe a 20-year-old woman with Turner syndrome presenting with a treatment-resistant rapid cycling bipolar disorder with cognitive impairment. She was diagnosed with anti-AMPA-receptor encephalitis. She showed marked improvement after starting memantine and valproic acid. *Conclusion.* This case description emphasises the importance of timely recognition of autoimmune limbic encephalitis in patients with psychiatric manifestations and a possible predisposition to autoimmune conditions, in order to rule out malignancy and to quickly initiate treatment.

## 1. Introduction

Turner syndrome (TS) is a common genetic disorder, affecting female individuals, resulting from the partial or complete absence of one sex chromosome and associated with reduced adult height and with gonadal digenesis, very frequently leading to infertility. Morbidity and mortality are increased in TS but average intellectual performance is within the normal range [1, 2]. No specific psychiatric condition has been traditionally related to TS, and TS is not mentioned in DSM-IV (APA 1994). In spite of this, a number of case reports described the association of TS with affective, schizoaffective, and schizophrenic psychoses [3]. TS is also associated with the presence of autoantibodies and with an increased risk of developing a wide repertoire of autoimmune disease.

Anti-*α*-amino-3-hydroxy-5-methyl-4-isoxazolepropionic acid (anti-AMPA) receptor encephalitis, characterized clinically by seizures, memory impairment, or psychosis, is one of the several types of newly recognized autoimmune encephalitis mediated by antibodies to neuronal surface proteins [4–6].

The absence of seizures and abnormalities on EEG and MR was reported in a few cases with anti-AMPA receptor encephalitis [7, 8], which might be explained by a possible noninflammatory mechanism in which AMPAR antibodies played a direct pathogenic role [7].

To the best of our knowledge, no data have been reported regarding autoimmune encephalitis in subjects affected by TS. In this paper we describe a case of a 20-year-old woman with TS who presented bipolar mood disorder, psychotic and other psychiatric symptoms, and anti-AMPA receptor encephalitis.

## 2. Case Presentation

### 2.1. Anamnestic Overview

Miss G. is a Caucasian, 20-year-old, not married woman, living with her parents, referred by her psychiatry to the Department of Psychiatry, Santa Chiara University Hospital, Pisa (Italy), for a consultation regarding her psychopathological condition, characterized by resistant continuous rapid cycles bipolar disorder. Miss G. has no family history of psychiatric disorder. She has no major medical problems related to the time of delivery, accomplished by a caesarean section because of oligohydramnios, with a birth weight of 2380 g. She had no alterations in motor cognitive development but had a delayed language acquisition (first words at 18 months).

In 2004, when she was 10 years old, because of her short stature and low weight, she underwent several investigations leading to Turner's syndrome diagnosis (45, XO/46X r (x) karyotype). She started growth hormone treatment. In the same year, she had her menarche and she showed a normal sexual development. Miss G. showed normal psychosocial adjustment during her early teen years. By the parents, she was described as brisk, sociable, and sportive child (she rides horses and used to dance as hobbies). Her IQ was not formally evaluated during the childhood because she had excellent school performance, as witnessed by the very high grades obtained in all the school exams.

In spring of 2008, at 14 years old, Miss G. showed the abrupt onset of an acute symptomatology characterized by anxiety, inner tension, language disabilities, stereotypies, and cognitive impairment. In few hours agitation, inattentiveness, confusion, mutism, bizarre behaviour, and paranoidism developed. Miss G. was promptly hospitalized and started several psychiatric consultations. During the hospitalization the clinical records indicated that Miss G. used to spend most of the time in aimless activities, staying lonely, smiling, and speaking alone (with herself). Her behavior and speech were disorganized, eye contact was absent, and mutism was the prevalent clinical feature. According to the psychiatrists that visited her during this hospitalization “psychotic symptoms and auditory hallucinations were present but not easily identifiable because of the language problems and severe speech disorganization.” She underwent neurological investigations (CT, MRI, and EEG that were normal) and cognitive assessment (IQ score of 74). Miss G. got the diagnosis of “Psychotic Disorder Not Otherwise Specified” and started treatment with antipsychotic medications (risperidone 3 mg/day later replaced by chlorpromazine 20 mg/day for inefficacy), discontinuing growth hormone therapy.

After the discharge from the hospital, in few weeks Miss G. experienced a progressively severe extrapyramidal symptomatology characterized by rigidity, tremors, and bradykinesia, associated with mutism, sedation, transient motor tic, stereotypies, and progressive deterioration of the cognitive functions. In few months she also developed hyperprolactinemia and amenorrhea.

From 2008 to 2011, Miss G. did not show clinical improvement and, through time, antipsychotic medications were progressively reduced. Extrapyramidal symptoms improved and menstrual period got back. At the same time, Miss G. parents started to notice that, during her premenstrual period, she showed a sudden change in her behaviour becoming hyperactive, restless, euphoric, and more energetic and sociable. Afterwards, from 2011 onwards, Miss G. continued experiencing mood, energy, and activity periodic fluctuations from sadness, low energy levels, and mutism to happiness, hyperactivity, and restlessness of variable duration, from 1 or 2 weeks to 1 or 2 months. Mood and activity switches where frequently observed during her periods. She was treated with several antidepressants and antipsychotics but she was not taking a continuous treatment because of inefficacy and frequent side effects. In few occasions when benzodiazepines were prescribed, Miss G. became frankly agitated and restlessness with disruptive behaviour.

At the beginning of 2014, a psychiatrist made a diagnosis of continuous rapid-cycling bipolar disorder and referred to our tertiary care Centre Miss G. for a clinical consultation. She was 20 years old, she was not taking psychiatric medications and she was still experiencing rapid cycling of mood and energy levels.

### 2.2. Clinical Presentation at Treatment Entry and Treatment Procedure

At the moment of the evaluation in our centre, Miss G. showed a condition of mild agitation and restlessness associated with euphoric mood and marked instability with daily fluctuations. The parents reported phases of motor retardation, mutism, and depressive mood alternating every few weeks with the current symptomatology. They also described a lack of functional autonomy and marked cognitive impairment with progressive memory loss.

Considering her treatments history we decided to avoid antidepressants, neuroleptics, and sedatives and we prescribed mood stabilisers (carbolithium 300 mg/day and valproic acid 600 mg/day). After 1 month Miss G. did not show any clinical improvement and parents referred to the onset of new side effects (e.g., enuresis). We decided to decrease the treatment posology (carbolithium 150 mg/day and valproic acid 600 mg/day) but side effects were still present. Miss G. showed excitement, hyperactivity, and restlessness particularly during her periods.

Because of the initial acute onset, the atypical course of the illness, the presence of neuropsychiatric symptoms, cognitive impairment, and rapid deterioration as well as the negative family history for mental disorders and the high prevalence of autoimmune diseases in patients with Turner syndrome, we hypothesized a possible autoimmune encephalitis [9]. Even though she had previous neurologic investigations that did not show signs of active encephalitis, we required some tests to look at anti-AMPA and N-Methyl-D-Aspartate (anti-NMDA) antibodies.

We found out that Miss G. was positive to anti-AMPA antibodies. We have excluded that the patient had a tumor by accurate physical and appropriate gynaecological examinations. We started treatment with memantine 10 mg and reduced the previous treatment prescription (we still prescribed 150 mg of valproic acid). Shortly after the onset of the treatment Miss G. showed a progressive improvement in her psychopathological condition. Her parents referred to the fact that she presented no more excitement episodes or hyperactivity. She showed an improvement in her cognition and scholar performance. In time, she was more sociable and started again to enjoy her free time riding horses and dancing. She had no side effects. We sent Miss G. to a Neurologic Consultation that confirmed our diagnostic hypothesis. After 6 months Miss G. is still taking 20 mg of memantine and she is stable from a psychopathological point of view, showing a marked improvement in global functioning.

## 3. Discussion

No specific psychiatric condition has been traditionally related to TS, and TS is not mentioned in DSM-IV-TR. In spite of this, a number of case reports described the association of TS with affective, schizoaffective, and schizophrenic psychoses. Cardoso and colleagues observed higher lifetime rate of mood disorders (but not psychiatric disorders in general) in women with TS compared to that reported in community-based samples, but comparable to that observed in gynecology clinic-based samples [10]. Other observations sustained the presence of affective disorders in general [11–13] and mania [14] in women with TS. A case report described psychosis as clinical features of 2 subjects affected by TS [15] and a schizophrenia-like illness and subsequently presenile dementia [16] has been reported in a 59-year-old patient with TS and normal intelligence; this patient developed also epilepsy. Other cases of TS with schizophrenia or affective psychosis have been reported [17].

TS is associated with the presence of autoantibodies and with an increased risk of developing a wide repertoire of autoimmune disease. The commonest diseases among these subjects are ulcerative colitis, Hashimoto thyroiditis, and, perhaps, type 1 diabetes mellitus. Coeliac disease, juvenile rheumatoid arthritis, Addison's disease, psoriasis, vitiligo, and alopecia areata have also been reported [18–25]. To the best of our knowledge, no data have been reported regarding autoimmune encephalitis in subjects affected by TS. Autoimmune diseases predominantly affect women, but the causes of the higher risk in women with TS remain unclear. Sex hormones, reproductive factors, fetal microchimerism, environmental factors, skewed X chromosome inactivation patterns, major defects in the sex chromosomes, and X chromosome gene dosage have all been proposed as being etiologically involved [7, 26, 27]. The higher risk of autoimmune diseases in women with Turner's syndrome has been suggested to be due in part to the haploinsufficiency of genes on the X chromosome [28]. However, other features of the TS phenotype may also be partly responsible for the elevated risk, possibly due to a complex interplay of genetic and environmental factors. The proinflammatory cytokines interleukin-6 and interleukin-8 and tumor necrosis factor seem to be upregulated in women with TS [29], and autoantibody positivity has been shown in a high proportion (57%) of cases [30]. It has also been hypothesized that, since autoimmune disorders are frequent in relatives of families of TS patients, abnormal gametogenesis and nondysfunctional events are due to abnormal autoimmune responsiveness [18, 31, 32]. Despite the fact that the strong association between TS and autoimmunity is well known and has been widely studied, the underlying immunopathogenic mechanism remains partially unexplained [33].

Miss G. case demonstrates that autoimmune encephalitis with prevalent psychiatric presentation may be misdiagnosed and mistreated as bipolar, schizoaffective, or schizophrenic disorders. Marked mood instability and psychotic features combined with rapidly progressive cognitive deterioration and resistance or abnormal response to the traditional treatments for bipolar disorder or psychoses are elements that may suggest the possibility of autoimmune encephalitis. Young age, female gender, and absence of family history for mental disorder should be also considered adjunctive diagnostic elements together with the presence of neuropsychiatric symptoms such as catatonic features, stereotypies, seizures, and speech disorders.

Autoimmune phenomena are being increasingly recognized as causes of encephalitis [34], and, as we have seen, TS is at high risk of developing autoimmune diseases. Autoimmune encephalitis is characterized by an acute onset of temporal lobe seizures, psychiatric features, and cognitive deficits [4, 8, 35]. The pathophysiology is typically mediated by autoantibodies targeting synaptic or intracellular autoantigens in association with a paraneoplastic or nonparaneoplastic origin [8]. As in addition to systemic autoimmune diseases associated with psychiatric manifestations (e.g., lupus) [36], more recently, patients with acute isolated psychosis were identified with synaptic autoimmune encephalitis [8, 37, 38]. Failure to appropriately diagnose the correct aetiology of an encephalitic syndrome can lead to significant morbidity and mortality, with possible chronicization. In the case report we presented, about 8 years have been spent to have the proper diagnosis from the onset of symptoms. This is because these patients are often erroneously diagnosed with refractory primary psychotic disorders, as has happened to our patient, delaying initiation of effective immune therapy or more specific therapy. In fact, the improper use of neuroleptics, antidepressants, and benzodiazepines has helped to worsen the course of the disorder of our patient. In addition, these patients have a particular sensitivity to the neurological side effects of these drugs [39].

The detection of the presence of anti-AMPAR antibodies in the serum of our patient, although having low titre (1 : 10), focused on the involvement of the glutamatergic system in the pathogenesis of the disorder. Glutamate-mediated excitotoxicity is the main pathological process taking place in many types of acute and chronic CNS diseases and injuries and glutamate is crucially needed for numerous key neuronal functions. Yet, excess glutamate causes massive neuronal death and brain damage by excitotoxicity, detrimental over activation of glutamate receptors. In recent years, it became clear that not only excess glutamate can cause massive brain damage, but also several types of antiglutamate receptor antibodies, that are present in the serum and CSF of subpopulations of patients with a kaleidoscope of human neurological diseases, can undoubtedly do so too, by inducing several very potent pathological effects in the CNS. Collectively, the family of antiglutamate receptor autoimmune antibodies seems to be the most widespread, potent, dangerous, and interesting antibrain autoimmune antibodies discovered up to now. There are no documented cases in the literature of anti-AMPA receptor encephalitis in individuals with TS.

We found that the clinical presentation of the patient with AMPA-ab was not restrict to that of limbic encephalitis, also presenting with rapid cycling-type mood instability and psychotic features. The normal findings in the brain MRI, TC, and EEG did not exclude the diagnosis. The absence of seizures and EEG and MR abnormalities has been reported in other cases with anti-AMPA receptor encephalitis [7, 8].

The rational use of memantine in this case is given by the lack of response to first-line treatment with stabilizers of an apparent rapid cycling bipolar disorder and by the exploitation of antiglutamatergic effect (nonspecific) in a patient whose pathogenesis appears to be related to a glutamatergic overtone secondary to the antibody-mediated decrease of AMPA receptors. In our patient, the absence of seizures and EEG and MR abnormalities suggested a possible noninflammatory mechanism in which AMPAR-Abs played a direct pathogenic role in glutamatergic transmission [7]. For this reason, in agreement with neurologists, we decided to use memantine, saving liquor examination and immunomodulatory therapies in the case of nonresponse.

Growing evidences show that memantine might be effective at preventing recurrences of both phases of bipolar disorder and in reducing the manic-like symptomatology associated with several neurological and psychiatric conditions [40–42]. It has been recently reported that memantine improves cognitive dysfunctions and increases hippocampal volume in euthymic bipolar patients [43]. Finally, we reported the results of a three-year naturalistic study of adding memantine to 30 treatment-resistant bipolar patients [42]. In this study memantine showed a long-term and progressive ability to prevent depressive and mania/hypomania recurrences, in patients who had been resistant to standard treatments for more than 3 years. Memantine decreases the duration of illness, the duration of new episodes, recurrence frequency, and symptomatology severity.

The developments in medicine in general and in psychiatry specifically, for example, autoimmune encephalitis, which have often been mentioned with an overlap and similarity to mood and psychotic disorders, clearly argue against the view that one size fits all or that there may be a single appropriate approach. Furthermore, the unspecificity of psychiatric symptoms is a well-established phenomenon, implicitly suggesting that the heterogeneity of the etiology is just the other side of one coin.

The prevalently psychiatric presentation of some of these autoimmune limbic encephalitis often leads patients to early psychiatric evaluation. For this reason it is very important to increase the limited knowledge among psychiatrists about these autoimmune neuropsychiatric diseases mimicking psychiatric syndromes, in particular severe mood disorders and schizophrenia. Protocols for assessment of patients presenting with the first episode of a psychotic disorder or an atypical course of bipolar disorder should always include medical screening, to exclude treatable organic causes. This is particularly important in those who present in an acutely confused state and are possibly delirious from an encephalopathic process as opposed to a functional psychosis or altered mood. Corroboration of experimental findings with the clinical picture is crucial. The possibility of a subgroup of antibody-positive patients with psychotic bipolar disorder highlights the importance of considering the complete clinical picture, including established investigations for organic disease, in order to differentiate between established antibody associated neuropsychiatric disease syndromes and purely psychiatric pathology.
